# Neuromuscular Alterations of Knee Extensors During Padel Match Play

**DOI:** 10.1002/ejsc.70263

**Published:** 2026-10-05

**Authors:** Antonin Jamotte, Marie‐Florine Michel, Machar Reid, Callum Beale, Olivier Girard

**Affiliations:** ^1^ School of Human Sciences (Exercise and Sport Science) University of Western Australia Perth Western Australia Australia; ^2^ Tennis Australia Melbourne Australia; ^3^ French Tennis Federation Stade Roland‐Garros Paris France

**Keywords:** explosive performance, intermittent activity, match demands, neuromuscular fatigue, racquet sports

## Abstract

Despite padel's growing popularity, its neuromuscular fatigue profile during prolonged match play remains poorly understood. This study examined the magnitude and etiology of neuromuscular alterations in the knee extensors using voluntary and evoked contractions during extended padel play. Sixteen trained padel players (14 males, 2 females) completed a 135‐min match. Neuromuscular assessments (maximal and explosive force, twitch and doublet responses) and vertical jump tests were conducted pre‐match (*Pre*) and after 45, 90, and 135 min (*Mid‐1*, *Mid‐2* and *Post*). Heart rate and match characteristics were continuously monitored. Maximal voluntary torque declined significantly across match play (−10% at *Post* vs. *Pre*, *p* < 0.05). Electrically evoked twitch and doublet torques decreased from *Mid‐1* onward (*p* < 0.05), while voluntary activation remained unchanged. Explosive force production and vertical jump performance were preserved (*p* > 0.05). Heart rate responses reflected moderate‐to‐high match intensity, with a progressive shift toward lower intensity zones despite stable technical activity patterns. Prolonged padel match play induces early peripheral neuromuscular fatigue without impairing voluntary activation or rapid force production. Although maximal knee extensor strength declined, players maintained explosive performance and jumping ability, suggesting effective compensation strategies during extended play.

## Highlights

- Prolonged padel induces early peripheral fatigue without central impairment
- Explosive force and jumping performance are preserved despite strength loss
- Players adapt match intensity through pacing rather than technical changes

**Abbreviations**
ANOVA: Analysis of variance
CMJ: Countermovement jump
D20: 20 Hz doublet
D80: 80 Hz doublet
EMG: Electromyography
HR: Heart rate
HRT: Half relaxation time
ICC: Intraclass correlation coefficient
KE: Knee extensor
MRTD: Maximal rate of torque development
MRTR: Maximal rate of torque relaxation
MVIC: Maximal voluntary isometric contraction
PPA: Peak‐to‐peak M‐wave amplitude
PPD: Peak‐to‐peak M‐wave duration
Pt: Peak torque
RMS: Root mean square
RPE: Rate of perceived exertion
RSI: Reactive Strength Index
RTD: Rate of torque development
SD: Standard deviation
SJ: Squat jump
TPT: Time to peak torque
VA: Voluntary activation
VL: Vastus lateralis

## 1 Introduction

Padel is one of the fastest‐growing sports worldwide, with participation expanding rapidly across multiple continents. Since 2010, the International Padel Federation has recognized 65 national federations and currently operates in over 90 countries (PadelFip: International Padel Federation 2025). As an intermittent sport involving moderate‐ to high‐intensity efforts (Castillo‐Rodríguez et al. 2014), padel offers various physical, psychological, and social benefits (Martínez et al. 2018). Previous research has documented its physical and physiological demands, including match temporal structure (Armstrong et al. 2023), in‐game characteristics (Conde‐Ripoll et al. 2024), player movement patterns (Ungureanu et al. 2022), and cardiovascular responses (Parraca et al. 2022). Padel matches are not time‐limited and typically last 45–180 min, with an average duration of ∼90 min (Ramón‐Llín et al. 2021). Competitive play requires frequent high‐intensity actions such as jumps, sprints, and changes of direction (Courel‐Ibáñez and Herrera‐Gálvez 2020). However, central and peripheral neuromuscular alterations may impair these actions, and despite increasing interest in padel's physical characteristics, its fatigue responses remain poorly characterized.

Neuromuscular fatigue is defined as an exercise‐induced decrement in the capacity to produce or maintain maximal force or power (Vøllestad 1997). It can result from both central (upstream the neuromuscular junction) and peripheral (i.e., contractile capacity of the muscle distant from the neuromuscular junction) mechanisms (Enoka 2008). In tennis, less pronounced neuromuscular impairment than that typically observed following prolonged endurance exercise (Millet and Lepers 2004) has been observed after 3 h of match play, with knee extensor (KE) maximal voluntary isometric contraction (MVIC) decreasing progressively by −1%, −5%, −6%, and −7% after 30, 60, 90, and 120 min, respectively, and reaching statistical significance after 150 (−13%) and 180 min (−9%) (Girard et al. 2008). This torque loss was accompanied by decreases in peak twitch torque (−8%) and the 20‐to‐80 Hz ratio (−12%), which is indicative of low‐frequency fatigue and impaired excitation–contraction coupling. In contrast, M‐wave amplitude and duration remained unchanged, suggesting preserved sarcolemmal excitability (Girard et al. 2008). In squash, a 1‐h match induced marked neuromuscular alterations, with reductions in KE MVIC (−16%) and voluntary activation (VA) (−7%), indicating a central component, whereas a decrease in peak torque (Pt) (−20%) reflects peripheral fatigue due to impaired excitation–contraction coupling (Girard et al. 2010). Padel differs from other racquet sports in several structural and tactical respects. It is played exclusively in doubles on a smaller enclosed court (10 × 20 m), where glass walls and metal fencing create less predictable ball trajectories and require rapid reactive repositioning (Miralles, Martínez‐Gallego, Guzmán, and Ramón‐Llin 2025a). These features contribute to higher effective playing time and more frequent net actions (volleys and overhead shots), but lower overall displacement and physical load than singles tennis (Armstrong et al. 2023). Consequently, the magnitude and central‐peripheral contribution to neuromuscular fatigue may differ from those reported in tennis and squash, thereby warranting direct investigation in padel.

In padel, explosive strength—the ability to rapidly generate force—is key for repeated high‐intensity actions on a small court, including directional changes, accelerations, lunges, jumps, and rapid positioning (Courel‐Ibáñez and Herrera‐Gálvez 2020). These actions rely heavily on force production within the first milliseconds following muscle activation, which traditional measures such as MVIC cannot capture and may therefore underestimate fatigue‐related functional impairments. For example, in badminton, explosive torque in both the plantar‐ and dorsi‐flexors decreased by up to 27% after two matches, whereas MVIC declined by only 7%–9%, highlighting the greater sensibility of explosive strength to fatigue (Girard et al. 2019). Moreover, fatigue‐induced changes in rate of force development vary between early (0–50 ms) and later (100–200 ms) contraction phases, suggesting the involvement of distinct neuromuscular mechanisms (Girard et al. 2019). Consequently, a comprehensive evaluation on fatigue in padel should include explosive strength measures across multiple time intervals, alongside traditional MVIC measures, to better reflect on‐court performance demands.

In racquet sports, effective fatigue monitoring is essential to minimize declines in physical performance. Despite the rapid expansion of padel, research on its neuromuscular fatigue profile remains scarce. To date, fatigue in padel has primarily been evaluated using reductions in countermovement jump (CMJ) height after a 90‐min match, revealing only modest declines in explosive power (Armstrong et al. 2023). However, CMJ height is a general measure and does not provide insight into the underlying mechanisms of fatigue.

The repeated high‐intensity actions characteristic of padel—including sprints, changes of direction, accelerations, decelerations, and jumps—place substantial demands on the neuromuscular system. Evidence from other racquet sports indicates that such intermittent activity can induce peripheral contractile fatigue, while prolonged match play may also compromise central drive (Girard and Millet 2009). Given the repeated requirement for rapid force generation during these actions, explosive force production may also be susceptible to fatigue. Therefore, this study examined neuromuscular responses to competitive padel play through repeated assessments, with a particular focus on KE maximal and explosive strength and the underlying central and peripheral mechanisms. We hypothesized that match play would induce significant reductions in maximal strength, with peripheral fatigue developing early and central fatigue emerging later. We further expected explosive strength, particularly in the early contraction phase, to be more sensitive to fatigue than maximal force.

## 2 Methods

### 2.1 Participants

Sixteen padel players (14 men and 2 women; age: 30.9 ± 6.2 y, body mass: 74.2 ± 14.6 kg, stature: 176.9 ± 8.0 cm) volunteered to participate after providing written informed consent. All participants were healthy, injury‐free adults who trained and competed in padel at least twice weekly, had a minimum of 3 years of playing experience, and competed at the regional level in Western Australia. None of the participants had a recent history (≤ 6 months) of myocardial infarction, bone fracture, or unstable cardiovascular, neuromuscular, or hypertensive conditions.

### 2.2 Experimental Design

The experimental design is illustrated in Figure 1. Participants first completed a familiarization session, followed by an experimental session conducted on an open‐air padel court. All sessions were held under comparable environmental conditions (∼18°C–22°C) and scheduled at the same time of day to minimize circadian influences.

**FIGURE 1:**
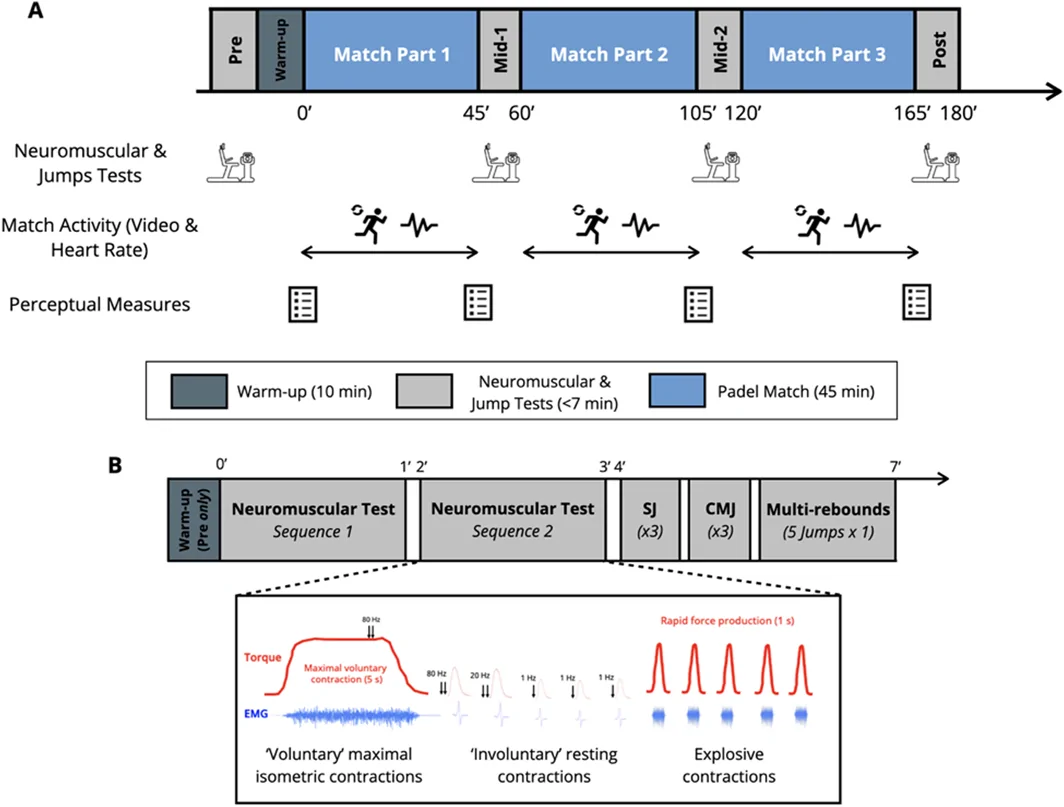
Experimental design and neuromuscular test battery. (A) Overview of the experimental protocol. After a 10‐min standardized warm‐up, participants completed a 135‐min padel match divided into three 45‐min blocks. Neuromuscular and jump tests (< 7 min per team of two players), as well as perceptual measures, were conducted before the match (*pre*) and immediately after each match block (*mid‐1*, *mid‐2*, *post*). Heart rate and match characteristics were continuously recorded during match play. (B) Detailed structure of the neuromuscular and jump test battery performed at each time point. The battery included: (i) maximal voluntary isometric contractions with superimposed electrical stimulations, (ii) electrically evoked contractions at rest (single twitches), and (iii) explosive contractions. Squat jumps (SJ), countermovement jumps (CMJ), and multirebound jumps were performed after the two neuromuscular test sequences.

### 2.3 Familiarization

Participants completed a single familiarization session that included: (1) a brief warm‐up with submaximal isometric contractions at 50%, 60%, 70%, 80%, and 90% of perceived maximal effort, followed by three 5‐s KE MVICs and 10 explosive contractions; (2) hands‐on practice with the electrical stimulation procedures; and (3) a passive isometric recruitment curve to identify the optimal stimulation intensity for eliciting maximal twitch torque and M‐wave amplitudes. Stimulation amplitude was progressively increased until a clear plateau in both responses was observed, with the optimal intensity defined as the lowest intensity at which this plateau was reached. Supramaximal stimulation was then set at 130% of this intensity to provide a margin above the plateau and account for potential minor day‐to‐day variability in nerve excitability. This intensity was maintained throughout subsequent experimental testing; (4) three trials of the neuromuscular test battery, each consisting of an MVIC with superimposed and resting electrical stimulations, followed by five explosive contractions (Figure 1); (5) three trials each of squat jump (SJ) and CMJ and two trials of the reactive strength index (RSI) protocol (multirebound jumps); and (6) a detailed briefing on study procedures, including explanations of perceptual scales (e.g., rating of perceived exertion [RPE], leg discomfort), equipment (e.g., GPS units, video camera), and the overall testing protocol.

### 2.4 Experimental Session

A minimum interval of 48 h separated the familiarization and experimental sessions. Participants completed a 10‐min standardized warm‐up consisting of 4 min of baseline rallies, 4 min of net play involving volleys and overhead shots, and 2 min of serves and returns. Participants then completed a 135‐min competitive match play protocol divided into three consecutive 45‐min blocks. Scoring was continuous until the end of each block. Games adhered to International Padel Federation rules (PadelFip: International Padel Federation 2025), except that changeover breaks were omitted to ensure that all participants accumulated equivalent playing time within each 45‐min block, thereby standardizing the neuromuscular assessment time points across pairs. Participants were paired according to playing level to avoid imbalance in match quality and to ensure that each athlete played on their habitual side of the court (left or right). Pairs remained unchanged, with no partner rotation throughout the protocol. A new set of three official balls was used for each match, with players retrieving balls between points. Although formal changeover breaks were omitted, players were permitted to drink water *ad libitum* (volume not quantified) after the neuromuscular assessment periods between match segments. Neuromuscular assessments and vertical jump tests were conducted at four time points: immediately before play (*Pre*) and after 45, 90, and 135 min (*Mid‐1*, *Mid‐2*, and *Post*), thereby capturing neuromuscular responses across match durations representative of real competitive padel, corresponding to short (∼45 min), average (∼90 min), and extended (∼135 min) competitive padel matches (Ramón‐Llín et al. 2021).

### 2.5 Neuromuscular Assessments

Participants were seated on an isometric ergometer with a strain gauge (Captels, St Mathieu de Treviers, France). Two straps positioned across the chest and pelvis‐minimized upper body movement, while participants crossed their arms over the shoulders to prevent compensatory motion. The trunk‐thigh angle was set at 90°, and the dynamometer axis aligned with the knee flexion‐extension axis. The lever arm cuff was positioned 2 cm above the lateral malleolus and secured using a Velcro strap. The knee angle was fixed at 80° flexion (0° = full extension). The ergometer was located next to the padel court to minimize the delay between playing and testing (Girard et al. 2008).

As illustrated in Figure 1, each neuromuscular test session consisted of two complete neuromuscular test batteries separated by 1 min of recovery. Each battery began with a ∼5 s MVIC of the right KE. Participants were instructed to gradually increase torque over ∼1 s, hold for 3–4 s, and then relax. Strong verbal encouragement was provided throughout, and the highest MVIC torque was retained for further analysis.

During each MVIC test sequence, six electrical stimuli were delivered 5 s apart: one 80 Hz doublet (D80) superimposed during the MVIC, followed by one D80, one 20 Hz doublet (D20), and three single twitches while fully relaxed (Figure 1). Voluntary activation (VA) was estimated using the twitch interpolation technique as: VA (%) = [1 − (superimposed D80/potentiated resting D80)] × 100, and was used as an index of central (neural) fatigue (Girard et al. 2008; Merton 1954).

The femoral nerve was electrically stimulated using a high‐voltage stimulator (Digitimer Ltd, Welwyn Garden City, England), delivering monophasic rectangular constant‐current pulses at up to 400 V with a pulse duration of 200 µs (Girard et al. 2008). A monopolar cathode ball electrode (0.5 cm diameter; Compex Motor Point Pen, Compex, Australia) was manually positioned within the femoral triangle, while a 50 cm^2^ rectangular anode (Medicompex, Ecublens, Switzerland) was placed within the gluteal fold. The stimulation site was marked on the skin to ensure consistent placement throughout the protocol.

Mechanical responses measured from single twitches included Pt, time to peak torque (TPT), half‐relaxation time (HRT), maximal rate of torque development (MRTD), and maximal rate of torque relaxation (MRTR) (Girard et al. 2008). These parameters were calculated as the average of three single twitches. For D80 and D20, values were taken from the best MVIC trial and the D20/D80 ratio was then calculated. Mechanical responses to electrical stimulation delivered to the relaxed muscle were used to assess peripheral contractile function. D20 and D80 provided measures of electrically evoked torque, while the D20/D80 ratio was used as an index of low‐frequency fatigue and alterations in excitation–contraction coupling.

Following the MVIC protocol, participants performed five explosive contractions, each separated by ∼2 s of rest. They were instructed to contract *“as fast as possible”* from a fully relaxed state for < 1 s. Contractions were considered valid when initiated from a stable, relaxed baseline without countermovement before torque onset. Five contractions were performed to ensure that at least three valid maximal efforts were obtained. Explosive torque production was quantified as rate of Torque development (RTD), calculated as the average slope of the torque–time curve from contraction onset to 30, 50, 100, and 200 ms (RTD0–30, RTD0–50, RTD0–100, and RTD0–200, respectively). Contraction onset was defined as the time point at which torque exceeded the baseline by > 1.5 Nm. The three best valid explosive contractions were retained and averaged for analysis.

Surface electromyographic (EMG) activity of the right *vastus lateralis* (VL) muscle was recorded using a pair of surface electrodes (EL503, Biopac Systems Inc., Goleta, CA, USA) fixed lengthwise over the muscle belly's midpoint. A reference electrode was positioned over the right knee. Low impedance between the two electrodes was obtained by abrading the skin with emery paper and cleaning with alcohol. Electrode placements were marked to ensure consistent placement, particularly if replacement was required due to sweating. EMG signals were amplified (30–500 Hz bandwidth), synchronized with torque recordings, and sampled at 2000 Hz (Biopac Student Lab 4.1, Goleta, CA, USA). Peak‐to‐peak M‐wave amplitude (PPA) and duration (PPD) were determined.

Valid MVIC torque, VA, and RMS activity measurements were available for 13 participants. Data from three participants were excluded because valid MVIC measurements could not be obtained due to suboptimal voluntary efforts. Consequently, reliable VA and RMS activity quantification was not possible because of inadequate superimposed twitch responses and/or EMG signal quality.

### 2.6 Vertical Jump Assessments

Vertical jump ability was assessed using a force platform (400+ Performance, Fitness Technology, Adelaide, Australia; 1000 Hz). Participants performed three vertical jump tests with hands placed on their hips to minimize upper limb contribution: (1) a SJ from a static semi‐squat position (∼90° knee flexion) maintained for ∼2 s without any preparatory movement; (2) a CMJ starting from standing, squatting down, and then extending the knees in one continuous movement; and (3) five consecutive multirebound jumps, aiming to rebound *“as high as possible”* while keeping the knees relatively stiff and minimizing ground contact time. Three trials were performed for SJ and CMJ, and one trial for the multirebound jumps with a 30‐s rest between jumps. Flight time and contact time were obtained from force platform recordings. Jump height was calculated for the SJ and CMJ, whereas RSI was calculated for multirebound jumps. For SJ and CMJ, the best trial was retained for analysis. For RSI, the average of three central values (excluding the highest and lowest values) was calculated from five attempts.

### 2.7 Exercise Characteristics

Heart rate (HR) was recorded continuously using a chest‐strap sensor (Vector S7, Catapult Sport, Melbourne, Australia). Individual maximal HR was assessed after completion of the experimental session using an incremental running test to exhaustion (20‐m shuttle run test; Léger and Lambert 1982). Exhaustion was defined as failure to maintain the required running pace for two consecutive stages, and HRmax was recorded as the highest HR reached during the test.

All matches were video‐recorded using an action camera (DJI Osmo Action 4, DJI, Nanshan, Shenzhen, China) and analyzed with custom‐developed Python software designed for notational analysis. Each shot was manually annotated to determine the technical and tactical characteristics, including the number of shots per rally, rally duration(s), rest time between rallies (s) and work‐to‐rest ratio. To assess the reliability of the match play analysis, intra‐ and inter‐rater reliability were determined by re‐coding 10% of randomly selected rallies after a 7‐day period by the same observer and a second independent analyst, respectively, both blinded to the original coding. Both observers were experienced sports scientists in racquet‐sport notational analysis. Intra‐observer reliability was rated as *excellent* (Landis and Koch 1977), with intraclass correlation coefficient (ICC) of 0.97 for rally duration and 0.94 for shot‐type classification. Inter‐observer reliability was also *excellent*, with ICC of 0.99 and 0.92 for rally duration and shot‐type classification, respectively, supporting the consistency of the coding framework.

Exercise‐related sensations, including RPE and leg discomfort, were assessed immediately after each time point (*Pre*, *Mid‐1*, *Mid‐2* and *Post*) using validated scales (Borg 1982, 1998).

### 2.8 Statistical Analysis

Sample size was determined a priori using G*Power (version 3.1, Heinrich Heine University Düsseldorf, Germany) for a repeated‐measures ANOVA (within‐subject factors). Based on a large effect size (*f* = 0.40) informed by previous racquet sport literature (Girard et al. 2008, 2010), *α* = 0.05, statistical power = 0.80, four measurement time points, and a correlation among repeated measures of 0.70, the minimum required sample was estimated to be 10 participants. All statistical analyses were performed using RStudio (RStudio, 1.1.463, RStudio Inc.). Data normality was verified using Shapiro–Wilk tests. Statistical significance was set at *p* < 0.05, and all results are presented as mean ± standard deviation (SD). Neuromuscular, jump and match‐related variables were analyzed using a one‐way repeated‐measures analysis of variance (ANOVA) with *Time* as the within‐subject factor (*Pre*, *Mid‐1*, *Mid‐2*, *Post*). Greenhouse–Geisser correction was used to account for sphericity violations. When a significant main effect was observed, pairwise post hoc comparisons were conducted using paired *t*‐tests with Bonferroni adjustment. For practical interpretation, individual percent changes from *Pre* were calculated as [(time‐point value−Pre value)/Pre value] × 100, averaged across participants, and reported as mean ± SD. Test–retest reproducibility between the familiarization and Pre sessions was assessed for the main neuromuscular variables using the standard error of measurement (SEM), coefficient of variation (CV%), and minimal detectable change at the 95% confidence level (MDC95). These metrics quantified measurement variability and are reported in Table S1.

## 3 Results

### 3.1 Torque Measurement

Neuromuscular outcomes assessed across the four time points (*Pre*, *Mid‐1*, *Mid‐2*, *Post*) are presented in Table 1 and Figure 2, 3. MVIC torque progressively declined throughout match play. Compared with *Pre* (199.6 ± 50.5 Nm), torque was significantly reduced at *Mid‐2* (178.3 ± 41.4 Nm; −10.0%; *p* < 0.05) and *Post* (178.0 ± 46.0 Nm; −10.4%; *p* < 0.05).

**TABLE 1:** Neuromuscular function during match play.

Variable	*Pre*	*Mid*‐*1*	*Mid*‐*2*	*Post*	Changes *pre‐post* (%)
Single twitch and M‐wave					
Pt (nm)	56.4 ± 23.4	50.9 ± 18.1	48.9 ± 18.2	50.2 ± 21.8	−9.0 ± 21.3
TPT (ms)	56.2 ± 19.5	48.2 ± 7.2	50.8 ± 7.5	52.1 ± 9.3	−1.1 ± 26.3
HRT (ms)	70.3 ± 19.9	56.5 ± 15.7**	58.4 ± 19.8***	49.4 ± 14.4***	−27.4 ± 17.0
MRTD (nm/ms)	1710 ± 741	1705 ± 585	1546 ± 599	1561 ± 755	−6.7 ± 27.4
MRTR (nm/ms)	965 ± 472	1097 ± 529	981 ± 579	1041 ± 585	+11.9 ± 36.6
PPA (mV)	9.35 ± 4.82	11.29 ± 4.21	10.09 ± 4.47	10.38 ± 5.05	+34.2 ± 80.5
PPD (ms)	10.85 ± 3.31	10.08 ± 2.04	9.92 ± 2.59	9.84 ± 2.52	−2.7 ± 35.1
Muscle activation and doublets					
RMS activity	0.41 ± 0.28	0.37 ± 0.15	0.31 ± 0.15	0.38 ± 0.23	+11.6 ± 49.4
VA (%)	89.0 ± 7.6	80.7 ± 20.8	86.6 ± 8.6	83.4 ± 9.2	−5.7 ± 13.2
D80	89.6 ± 26.4	85.0 ± 27.1	82.7 ± 29.1	88.1 ± 28.2	−0.6 ± 19.3
D20	84.2 ± 30.1	78.2 ± 28.8	73.0 ± 29.0	79.5 ± 26.6	+0.8 ± 37.5
Explosive strength					
Explosive_peak_ (nm)	185.4 ± 51.1	176.0 ± 47.5	191.2 ± 48.8	180.1 ± 45.9	−1.4 ± 16.0

*Note:* All variables (*n* = 16), except for RMS activity and VA (*n* = 13). Pre: *pre*‐match; *Mid*‐*1*, *Mid*‐*2*, *Post*: 45, 90, and 135 min into match play. Values are mean ± SD.

Abbreviations: D20, doublets at 20 Hz; D80, doublets at 80 Hz; HRT, half relaxation time; MRTD, maximal rate of torque development; MRTR, maximal rate of torque relaxation; PPA, Peak‐to‐peak M‐wave amplitude; PPD, Peak‐to‐peak M‐wave duration; Pt, Peak torque; RMS, root mean square activity; TPT, time to peak torque; VA, voluntary activation.

**(*p* < 0.01), ***(*p* < 0.001) denotes a significant difference from *Pre*.

**FIGURE 2:**
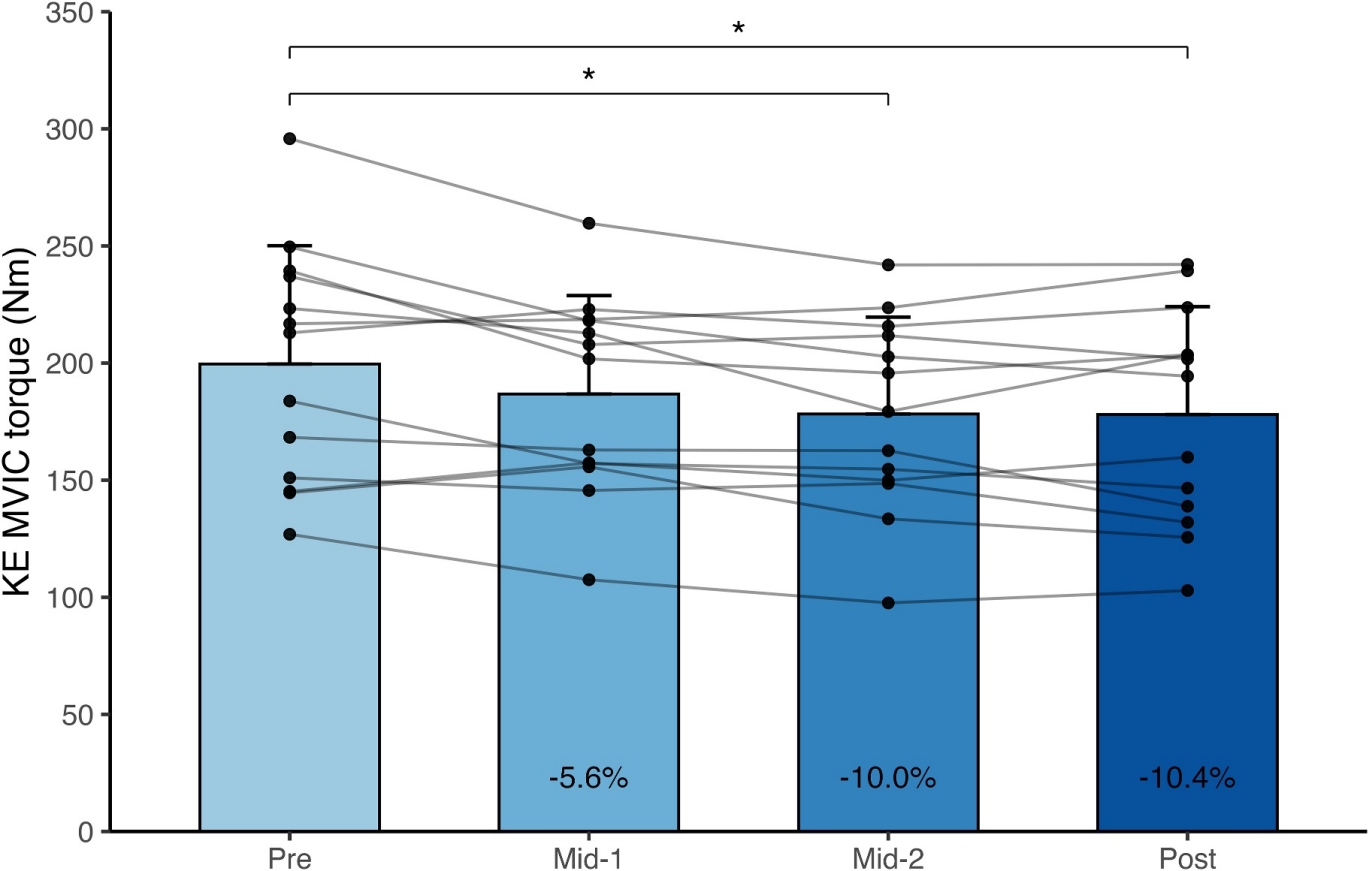
Isometric maximal voluntary contraction of knee extensors (KE MVIC) measured pre‐match (*Pre*) and after 45, 90, and 135 min of play (*mid‐1*, *mid‐2* and *post*) (*n* = 13). *p < 0.05, denotes a significant difference versus *pre.*

**FIGURE 3:**
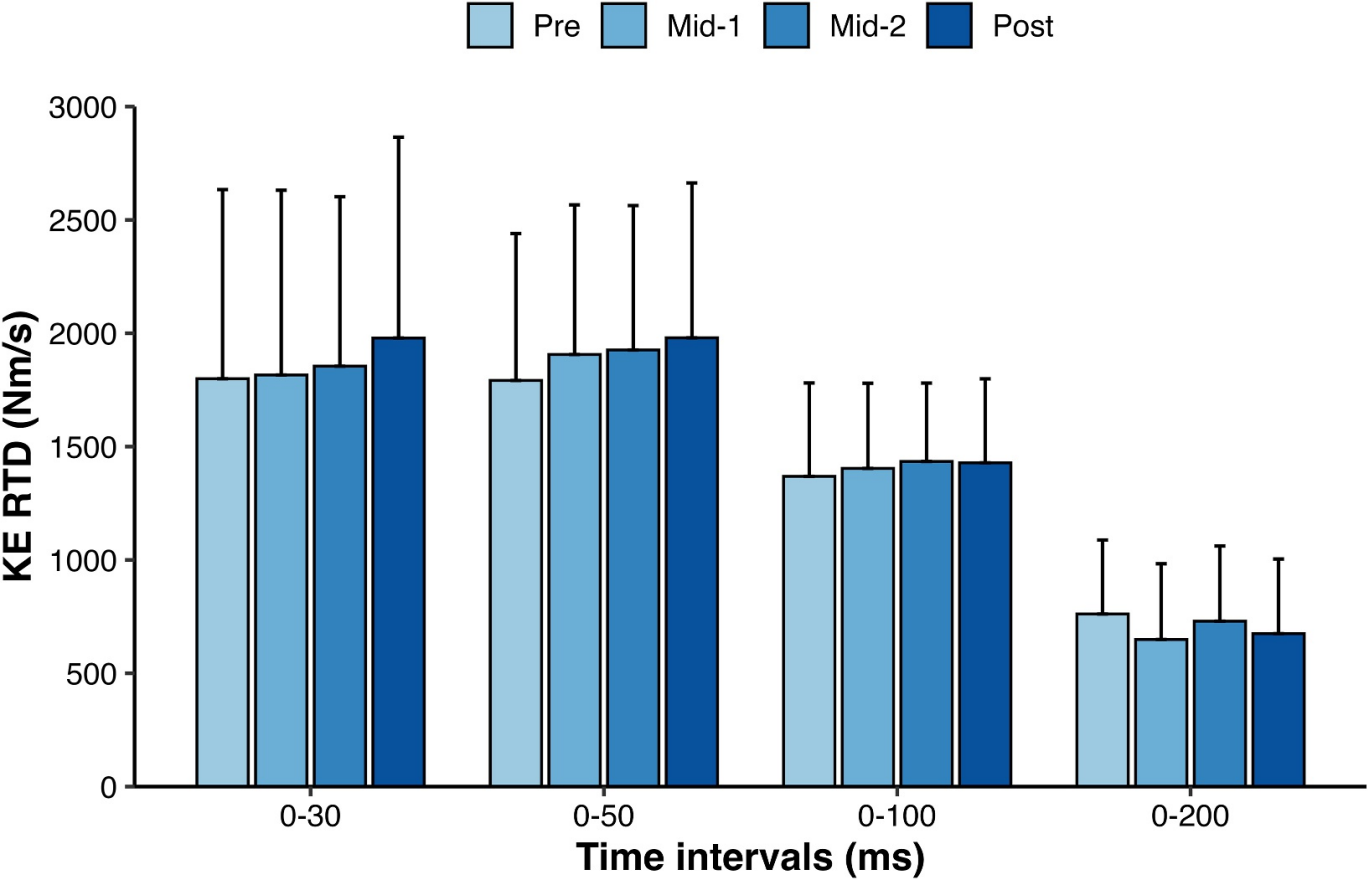
Rate of torque development (RTD) during explosive isometric knee extensions obtained at 0–30, −50, −100 and −200 ms pre‐match (*pre*) and after 45, 90, and 135 min of play (*mid‐1*, *mid‐2* and *post*) (*n* = 16).

No time‐dependent changes were observed in VA, RMS activity, electrically evoked contractile properties (D80 and D20), and explosive torque‐production capacity at any time intervals (Table 1). Single twitch parameters also remained largely unchanged, except for a significant reduction in HRT (*Pre*: 70.3 ± 19.9; *Mid‐1*: 56.5 ± 15.7 [*p* < 0.01]; *Mid‐2*: 58.4 ± 19.8 [*p* < 0.001]; *Post*: 49.4 ± 14.4 ms [*p* < 0.001]), corresponding to a −27.4 ± 17.0% *Pre*‐to‐*Post* change.

### 3.2 Vertical Jump Ability

Jump height for both SJ and CMJ as well as RSI during multirebound jumps did not significantly change (all *p* > 0.05, Table 2).

**TABLE 2:** Jump performance metrics during match play.

Variable	*Pre*	*Mid*‐*1*	*Mid*‐*2*	*Post*	Changes *pre‐post* (%)
Height_SJ_ (cm)	30.4 ± 5.2	32.3 ± 5.9	32. 8 ± 6.3	33.3 ± 6.7	+9.3 ± 10.4
Height_CMJ_ (cm)	33.4 ± 6.1	34.7 ± 5.9	35.8 ± 6.5	35.4 ± 7.2	+6.0 ± 9.3
RSI_Multirebound_	0.87 ± 0.27	0.90 ± 0.26	0.93 ± 0.28	0.94 ± 0.30	+11.0 ± 26.2

*Note:* Mean ± SD values for squat jump height (Height_SJ_), countermovement jump height (Height_CMJ_), and reactive strength index from multirebound jumps (RSI_Multirebound_) at baseline (*Pre*) and following 45, 90, and 135 min of match play (*Mid*‐*1*, *Mid*‐*2*, and *Post*, respectively).

### 3.3 Exercise Characteristics

Exercise characteristics across the three match segments are presented in Table 3. HR_mean_ reflected moderate‐to‐high exercise intensity, decreasing slightly but significantly over time (0–45 min: 143.3 ± 17.9 bpm; 90–135 min: 133.9 ± 18.1 bpm, *p* < 0.01). A similar pattern was noted for HR_peak_ (0–45 min: 171.4 ± 16.1 bpm; 90–135 min: 163.9 ± 16.3 bpm, *p* < 0.01). Based on HR zone distribution, time spent in lower intensity zones (50%–70% HR_max_) increased significantly (*p* < 0.05), whereas no significant changes were observed in the higher intensity zones (≥ 80% HR_max_), despite lower mean values across match segments.

**TABLE 3:** Match play characteristics and heart rate responses during match play.

Variable	0–45 min	45–90 min	90–135 min
Stroke type (*n* = 16)			
Forehand	74.8 ± 38.7	69.6 ± 33.3	71.4 ± 24.0
Backhand	53.0 ± 15.9	48.8 ± 13.7	46.2 ± 16.0
Forehand volley	19.2 ± 7.9	22.8 ± 7.5	16.4 ± 7.4
Backhand volley	19.4 ± 11.0	20.4 ± 7.5	15.5 ± 10.1
Overhead	33.4 ± 12.9	30.1 ± 6.7	29.6 ± 11.2
Rallies and time intervals (*n* = 16)			
Average number of shots per rally	4.9 ± 0.8	5.0 ± 0.4	4.9 ± 0.6
Average rally duration (s)	9.1 ± 0.6	8.6 ± 0.5	8.7 ± 0.7***
Rest time between rallies (s)	10.7 ± 0.6	11.9 ± 1.6	12.7 ± 1.1***
Play:Rest ratio	0.85 ± 0.05	0.74 ± 0.13	0.69 ± 0.06***
Heart rate zones (*n* = 14)			
50%–60%	11.0 ± 18.0	16.7 ± 23.4	19.3 ± 21.0*
60%–70%	24.2 ± 20.1	32.6 ± 21.2	36.8 ± 22.9*
70%–80%	34.5 ± 19.4	31.1 ± 21.4	25.7 ± 20.1
80%–90%	21.3 ± 21.9	14.7 ± 18.7	15.4 ± 19.8
90%–100%	8.6 ± 22.0	4.2 ± 14.5	2.6 ± 9.2
Heart rate (bpm) (*n* = 14)			
Mean heart rate	143.3 ± 17.9	135.7 ± 18.4	133.9 ± 18.1**
Peak heart rate	171.4 ± 16.1	167.5 ± 14.7	163.9 ± 16.3**

*Note:* Values are mean ± SD.

*(*p* < 0.05); **(*p* < 0.01); ***(*p* < 0.001) denote significant differences between 0–45 min and 90–135 min.

Technical activity patterns remained stable throughout the match, with no significant changes in total stroke count or in the distribution of forehand, backhand, volley, and overhead shots. Rally characteristics were also largely unchanged, with no differences in shot number or rally duration over time. However, rest time between rallies increased significantly across match play (0–45 min: 10.7 ± 0.6 s; 90–135 min: 12.7 ± 1.1 s; *p* < 0.001). This increase was accompanied by a reduced work‐to‐rest ratio during the final match segment (0–45 min: 0.85 ± 0.05; 90–135 min: 0.69 ± 0.06; *p* < 0.001), indicating a progressive shift toward longer recovery intervals as the match progressed.

### 3.4 Perceptual Responses

Compared to *Pre* (1.3 ± 0.6), RPE gradually increased at *Mid‐1* (2.8 ± 0.8, *p* < 0.001), *Mid‐2* (3.8 ± 1.2, *p* < 0.001), and *Post* (4.5 ± 1.4, *p* < 0.001). Similarly, limb discomfort increased from *Pre* (1.4 ± 0.8) to *Mid‐1* (2.8 ± 1.0, *p* < 0.001), *Mid‐2* (3.7 ± 1.1, *p* < 0.001), and *Post* (4.5 ± 1.5, *p* < 0.001).

## 4 Discussion

### 4.1 Summary of Main Findings

We tested the hypothesis that padel match play would reduce KE force production, with early peripheral fatigue and later central fatigue, and that early‐phase explosive strength would be more sensitive to fatigue than MVIC torque. Contrary to our hypothesis, MVIC torque decline was accompanied by progressive reductions in electrically evoked torques but occurred without changes in VA, while explosive force across all contraction time intervals remained unchanged. Padel match play induces peripheral fatigue without impairing voluntary explosive force production, suggesting that players may preserve rapid force‐generating capacity despite reductions in maximal strength.

### 4.2 Maximal Strength

We found that KE MVIC torque decreased by ∼10% after 135 min of padel play, a smaller magnitude than that reported in badminton (−12% after 1 h) (Lin et al. 2023), tennis (−13% after 3 h) (Girard et al. 2006), and squash (−16% after 1 h) (Girard et al. 2010). Notably, most of the force reduction emerged within the first 45 min, unlike in tennis where strength declined progressively over a 3‐h simulation with assessments every 30 min (Girard et al. 2008). These cross‐sport comparisons should be considered with caution, as differences in match duration, testing protocols (actual *vs*. effective playing time), playing surfaces, and the doubles format of padel likely influence neuromuscular demands compared with singles‐based racquet sports (Girard and Millet 2009).

### 4.3 Peripheral Fatigue

Quadriceps twitch torque declined by ∼10% early in play (*Mid‐1*) and showed no further meaningful alterations thereafter. This reduction may reflect subtle alterations in contractile function involving changes in Ca^2+^ handling and metabolite accumulation (Allen et al. 2008; Calderón et al. 2014), although this interpretation remains speculative. Comparable, though often larger, contractility deficits (−10%–15%) have been reported in other racquet sports (Girard et al. 2010; Lin et al. 2023). Notably, HRT progressively decreased throughout match play, indicating faster muscle relaxation. This response differs from the prolonged relaxation commonly associated with skeletal muscle fatigue and impaired Ca^2+^ handling (Allen et al. 2008), although relaxation responses may vary according to the exercise characteristics and muscle studied (Booth et al. 1997). Altogether, the reduction in MVIC cannot be attributed unequivocally to peripheral contractile fatigue, and its underlying mechanisms remain uncertain.

### 4.4 Central Fatigue

No time‐dependent changes were observed in surface EMG activity (RMS values) and muscle activation capacity measured using the twitch interpolation technique. However, because M‐wave responses were not calculated, RMS could not be normalized and should be interpreted cautiously as an indicator of neural drive (Girard et al. 2018). The unchanged VA suggests that central fatigue did not play a major role in the observed KE strength deficits—unlike in other racquet sports where it has been more prominent (Girard and Millet 2009). VA was relatively low at baseline (∼84%) with substantial inter‐individual variability, consistent with reports in tennis players (∼85%) (Girard et al. 2008). Importantly, our testing conformed to recent Delphi consensus guidelines, including the use of doublet stimuli, self‐adhesive electrodes, familiarization, feedback during contractions, supramaximal stimulation, and manual triggering (Osborne et al. 2023). Taken together, these findings provide no evidence of substantial central activation impairment during match play, although the absence of RMS/M‐wave normalization limits conclusions regarding changes in neural drive.

### 4.5 Explosive Strength

To our knowledge, this is the first study to report no match‐induced changes in rapid muscle force production at any time point, despite clear MVIC strength deficits. This contrasts with tennis findings, where contractile RTD declined across all intervals after 2 h of play (Girard et al. 2014), and successive badminton matches (2 × 35, 15 min of rest), where explosive strength losses exceeded MVIC reductions (Girard et al. 2019). Such discrepancies may reflect sport‐specific demands, including court dimensions and match format (doubles *vs*. singles), all of which influence movement intensity and neuromuscular load. Importantly, explosive and maximal strength were assessed using separate contraction types rather than using the common *“hard‐and‐fast”* instruction. This distinction matters, as ballistic (*“fast‐only”*) contractions better capture motor neuron discharge behavior and agonist activation (Duchateau and Baudry 2014), reducing the likelihood of underestimating explosive force. Nevertheless, padel match play did not impair the rapid force production capacity of the KE.

### 4.6 Match Characteristics and Intensity

The performance indices and physiological demands observed were consistent with previous data in similarly skilled padel players. Prior work has shown that padel involves frequent volleys and overheads, with average HR at ∼70%–90% HR_max_ (Mellado‐Arbelo and Baiget 2022), which was also evident here. A novel finding was that across the three 45‐min match segments, players spent progressively less time in higher‐intensity zones (> 70% HR_max_) and more time at lower intensities (50%–70% HR_max_), despite no changes in shot‐type distribution. Thus, players maintained shot selection patterns while gradually shifting toward lower‐intensity effort without altering rally characteristics. These results contrast with Marcos‐Rivero et al. (2025), who reported progressive increases in physiological responses in amateur players, with mean and peak HR and time in higher‐intensity zones rising from the first to third set of ∼60‐min matches. A possible explanation is that real competitive contexts elicit greater sympathetic activation during later sets when decisive points demand greater effort, whereas our simulated match did not replicate these conditions. Alternatively, players may have adjusted their activity patterns to manage fatigue and cardiovascular strain (Girard and Millet 2009). However, the mechanisms underlying the progressive reduction in HR cannot be established from the present data, and factors such as changes in match intensity, recovery between rallies, and hydration status may have contributed. While such adjustments may have contributed to variability in fatigue responses, they likely reflect ecologically valid behavioral adaptations in padel match play. Future studies using GPS‐derived movement metrics are warranted to confirm whether players progressively reduce total and high‐intensity court displacements (i.e., acceleration and decelerations) during prolonged padel play (Armstrong et al. 2023).

### 4.7 Exercise‐Related Sensations

According to Enoka and Duchateau (2016), fatigue encompasses both perceived and performance fatigability. While neuromuscular assessments via percutaneous nerve stimulation (e.g., VA and contractile responses) primarily capture performance fatigability, they should ideally be complemented by subjective measures of exercise‐related sensations. Using CR‐10 scales, perceived exertion and leg discomfort increased progressively during the match but remained within moderate levels, consistent with previous padel research (Armstrong et al. 2023). A limitation of our study is that mental freshness was not controlled prior to the match protocol, which may have influenced perceptual responses. Acute mental fatigue has been shown to reduce the accuracy and speed of padel‐specific strokes and impair Stroop and vigilance performance in youth‐elite male players, emphasizing the need to monitor and manage mental fatigue in this sport (Díaz‐García et al. 2024).

### 4.8 Vertical Jump Ability

The padel match protocol did not affect vertical jump ability, whether assessed by jump height (SJ, CMJ) or leg stiffness (RSI). Similar findings have been reported after simulated matches lasting ∼50–75 min (Marcos‐Rivero et al. 2025; Pradas et al. 2021), although reductions in CMJ height (−4.6%) and/or leg stiffness (−6%) have been observed after 60 min elsewhere (Armstrong et al. 2023). Players may alter jump mechanics when fatigued to preserve jump height (Gathercole et al. 2015). Moreover, our neuromuscular tests targeted isometric KE function, while jump performance reflected dynamic multi‐joint actions, which may help explain the lack of correspondence. Indeed, Márquez et al. (2023) demonstrated that jump potentiation can coexist with peripheral fatigue of the KE, suggesting that preserved or improved jump height does not preclude underlying neuromuscular impairment. Jump height remained unchanged despite reductions in maximal KE strength, suggesting that jump height alone does not capture all neuromuscular alterations occurring during prolonged padel play.

### 4.9 Limitations and Additional Considerations

A strength of this study was the assessment of neuromuscular function during both familiarization and pre‐match testing, followed by repeated evaluations after each of three successive 45‐min match segments, rather than relying solely on pre‐ versus post‐match comparisons. Nevertheless, some features of the protocol may not fully replicate real competitive conditions. Changeover breaks were deliberately omitted to standardize playing time across assessment blocks, while the fixed protocol structure, despite capturing short, average, and extended match durations typical of competitive padel, may not reflect the natural variability in match duration. Moreover, because participants were aware of the fixed 135‐min match duration, anticipatory pacing may have influenced effort distribution and potentially attenuated the magnitude of neuromuscular fatigue observed during the protocol. The absence of competitive stress, including the psychological pressure associated with a decisive scoreline or tournament context, may have influenced pacing behavior and physiological responses. These factors should be considered when extrapolating the present findings to real match play.

In addition, we did not examine the recovery time course of neuromuscular alterations in the minutes to hours following the match protocol, which is particularly relevant when multiple matches are played in close succession. For instance, KE MVIC torque has been shown to decrease further from immediately post‐to 1 h after a 60‐min badminton match (Lin et al. 2023). Playing two consecutive matches in a World Padel Tour competition can increase external load—particularly accelerations, decelerations, and maximum speeds—during the first game, potentially contributing to fatigue and reduced performance in subsequent matches (Miralles, Martínez‐Gallego, Guzmán, and Ramón‐Llin 2025b). Successive matches in professional padel have also been shown to impair overall performance and various physical qualities, including reaction time, explosiveness, and balance. Future research should investigate the cumulative effects of multiple games played within the same day and across consecutive days.

Neuromuscular fatigue was assessed only in the quadriceps. Although other lower‐limb muscles (hamstrings, calves, hip adductors/abductors) contribute to padel actions (Cocco et al. 2024) and may also experience fatigue, measuring them was not feasible. The quadriceps were selected because of their key role in explosive and stabilizing movements in padel (i.e., forward propulsion, jumping, and impact absorption) and their demonstrated susceptibility to match‐induced fatigue in racquet sports (Girard and Millet 2009). Fatigue may also affect dynamic balance. Indeed, Bourara et al. (2023) reported reduced anterior reach distance in elite padel players following exhaustive exercise, potentially increasing injury risk and highlighting the importance of assessing fatigue tolerance in muscles critical for balance.

Although the sample comprised competitive adult padel players, the findings may not generalize to beginners, elite players, younger or older cohorts, or a broader female population, as only two female participants were included. Even within this relatively homogeneous sample, large inter‐individual differences were observed. The inherently unpredictable nature of competitive padel, shaped by tactical decisions of partners and opponents, leads to substantial variability in match activity (Mellado‐Arbelo and Baiget 2022). Consequently, the magnitude of fatigue and timing of neuromuscular adjustments varied between players, as illustrated by MVIC torque. Indeed, some showed no change, whereas others experienced substantial declines (up to 25%), despite a modest group mean decrease averaging ∼10% (Figure 2). Patterns also differed temporally, with some players declining early, others progressively, and some only at match end. The two female participants showed MVIC responses broadly consistent with the overall group pattern and did not disproportionately contribute to the observed inter‐individual variability.

### 4.10 Practical Applications

Prolonged padel match play was associated with reductions in maximal KE strength, whereas no significant changes were observed in explosive strength and jump performance. These findings suggest that maintaining maximal lower‐limb strength and fatigue resistance, particularly of the quadriceps, may be relevant for prolonged match play. Caution is warranted when using jump height alone as a surrogate measure of neuromuscular readiness, as unchanged jump performance may co‐exist with reductions in maximal force production. Players also exhibited lower cardiovascular intensities and longer between‐rally recovery periods over time; however, whether these changes reflect pacing, fatigue, or other physiological or behavioral factors remains unclear.

## 5 Conclusion

Neuromuscular fatigue during a 135‐min competitive padel match was characterized through repeated assessments at 45, 90, and 135 min, examining maximal and explosive strength and their underlying central and peripheral mechanisms. MVIC declined early (by 45 min) and was accompanied by alterations in KE contractile properties, while explosive force, muscle activation, and vertical jump performance remained unchanged.

## Author Contributions

All authors conceived and designed the research. M.‐F.M. and A.J. conducted the experiments. M.‐F.M. and A.J. contributed new analytical tools. M.F.M. and A.J. analyzed data. M.‐F.M., A.J., O.G., and M.R. wrote the manuscript. All authors have revised the manuscript. All authors have read and approved the manuscript.

## Funding

The authors have nothing to report.

## Ethics Statement

The study was approved by the Human Research Ethics Committee of The University of Western Australia (2025/ET000215) and was conducted in compliance with the 1964 declaration of Helsinki.

## Consent

Written informed consent was obtained from all participants prior to participation in the study.

## Conflicts of Interest

The authors declare no conflicts of interest.

## Permission to Reproduce Material From Other Sources

The authors have nothing to report.

## Supporting information


**Table S1:** Test–retest reproducibility of the main neuromuscular variables between the familiarization and pre sessions.

## Data Availability

The data that support the findings of this study are available from the corresponding author upon reasonable request.
