# Supplementary material for: Neuromuscular Alterations of Knee Extensors During Padel Match Play
Source: Eur J Sport Sci. 2026 Oct 5;26(11):e70263. doi: 10.1002/ejsc.70263 (PMC13635870; doi:10.1002/ejsc.70263)
Supplement: Supplementary file 1 [file EJSC-26-e70263-s001.docx]

Supplementary Table 1 Test-retest reproducibility of the main neuromuscular variables between the familiarisation and Pre sessions.

| **Variable** | ***Fam*** | ***Pre*** | **SEM** | **CV (%)** | **MDC95** |
| --- | --- | --- | --- | --- | --- |
| MVIC torque (Nm) | 201.2 ± 46.4 | 199.6 ± 50.5 | 19.0 | 9.5 | 52.8 |
| VA (%) | 84.9 ± 7.4 | 89.0 ± 7.6 | 6.63 | 7.6 | 18.4 |
| Explosive_peak_ (Nm) | 177.9 ± 57.7 | 185.4 ± 51.1 | 27.8 | 15.3 | 77.1 |
| Pt (Nm) | 57.2 ± 22.0 | 56.4 ± 23.4 | 13.7 | 24.2 | 38.1 |
| D80 (Nm) | 87.9 ± 29.2 | 89.6 ± 26.4 | 19.5 | 21.9 | 54.0 |
| D20 (Nm) | 81.7 ± 27.8 | 84.2 ± 30.1 | 19.6 | 23.6 | 54.3 |
| RMS activity | 0.27 ± 0.12 | 0.41 ± 0.28 | 0.17 | 49.2 | 0.46 |

*All variables (n = 16), except for MVIC torque, VA and RMS activity (n = 13). Values are mean ± SD.*

*Fam: Familiarization; Pre: pre-match; SEM: standard error of measurement; CV: coefficient of variation; MDC95: minimal detectable change at the 95% confidence level.*

*Isometric maximal voluntary contraction (MVIC) torque, voluntary activation (VA), peak torque (Pt), doublets at 80 Hz (D80) and 20 Hz (D20) and root mean square (RMS) activity.*
